# Methylene Blue Spray for Identification of Parathyroid Glands During Thyroidectomy

**DOI:** 10.7759/cureus.11569

**Published:** 2020-11-19

**Authors:** Sherif Monib, Abdullah Mohamed, Mohamed I Abdelaziz

**Affiliations:** 1 Breast Surgery, West Hertfordshire Hospitals NHS Trust, St Albans and Watford General Hospitals, London, GBR; 2 Surgery, Fayoum University Hospitals, Fayoum, EGY

**Keywords:** thyroidectomy, parathyroid gland, methylene blue spray, hypocalcaemia

## Abstract

Background: Hypocalcaemia is a common delayed complication after thyroidectomy. Several studies have identified risk factors and possible ways to prevent post-thyroidectomy hypocalcemia. The purpose of our study is to evaluate the effectiveness of an intraoperative methylene blue spray to identify parathyroid glands during thyroidectomy.

Materials and methods: We have conducted a prospective non-randomised cohort study with 50 patients who underwent hemithyroidectomy or total thyroidectomy between January 2019 and January 2020. During thyroidectomy, 1 ml (10 mg) of 1% methylene blue was sprayed over the parathyroid glands, the inferior thyroid artery, and the recurrent laryngeal nerve.

Results: Our study included 50 patients with ages ranging from 18 to 80 years old (43.0±9.7). We were able to identify the parathyroid glands with the intraoperative methylene blue spray in 82% of cases, with no significant postoperative complications.

Conclusion: Our study concludes that the methylene blue spray is a safe, feasible, and effective technique to identify parathyroid glands.

## Introduction

Parathyroid glands are responsible for calcium homeostasis. Any injury to the parathyroid glands or their blood supplies during thyroidectomy might lead to permanent hypocalcaemia in 3.3% of patients or transient hypocalcaemia in 33.3% [1]. The treatment for hypocalcaemia requires calcium therapy for over six months after surgery. Unfortunately, hypocalcaemia does not only affect the mental and physical state of patients but also lengthens their hospital stay [1]. Hypocalcaemia can be diagnosed through perioral or finger numbness, muscle spasms, cramping, seizures, or cardiac arrhythmia, and in severe cases a positive Chvostek’s sign. The condition can be treated initially with calcium supplements and vitamin D [2].

The prevention of injuries to the parathyroid thyroid glands during thyroid surgery relies on the correct identification of the glands, through anatomical landmarks, a partial biopsy of the glands for pathological examination [3], optical coherence tomography during surgery [4], parathyroid specific luminescence [5], fine needle aspiration for an analysis of parathyroid, and measurement of parathyroid hormone levels in blood [6] or an intravenous methylene blue injection [7].

Methylene blue was first introduced in 1876 by Heinrich Caro [8]. Also known as methylthioninium chloride, this thiazine dye converts the ferric iron in haemoglobins to ferrous iron. Methylene blue was mainly used to treat methemoglobinemia, urinary tract infections, and cyanide poisoning, although it is no longer recommended for these purposes. Side effects include headache, confusion, vomiting, shortness of breath, high blood pressure, serotonin syndrome, haemolysis, and allergic reactions. Methylene blue injections may often colour the urine, sweat, and stool blue or green [9,10]. 

Methylene blue dye is readily available, but not without its complications, especially if injected intravenously. Therefore, we aimed to evaluate the diagnostic value of using a methylene blue spray to identify parathyroid glands during thyroidectomy to help avoid injuries and future complications.

We aimed to evaluate the safety and reliability of the methylene blue spray technique in the identification of parathyroid glands as a cheap modality which does not entail the use of costly equipment or require further training to decrease post-thyroidectomy hypocalcaemia.

## Materials and methods

Ethical approval was obtained from the Fayoum University Hospital Surgical Department Ethics Committee prior to commencing the study.

We conducted a prospective non-randomised study of 50 patients who underwent hemithyroidectomy or total thyroidectomy in the Fayoum University Hospital between January 2019 to January 2020. Seventy percent (N=35) were females, and 30% (N=15) were males, ages ranged from 18 to 80 (43.0±9.7).

We included patients aged 18 years or older, who were diagnosed with simple or toxic nodular goitres requiring surgery, and who fit the criteria for general anaesthesia. We excluded patients known to have methylene blue allergies, glucose-6-phosphate dehydrogenase deficiency, chronic kidney diseases, recurrent diseases, patients who received monoamine oxidase (serotonin syndrome), and patients unwilling to take part in the study.

Preoperative evaluation included a review of medical history with special attention to medications, previous surgeries and manifestations suggestive of hypothyroidism, hyperthyroidism, or thyroid malignancies. Subsequently, we performed a clinical examination to identify signs of hypothyroidism, hyperthyroidism, thyroid malignancies, or retrosternal extension of goitre.

Laboratory investigations included routine preoperative blood tests 24h before surgery, including serum ionised calcium (SiCa) levels, parathyroid hormone (PTH) levels and thyroid profile.

All patients underwent a baseline neck ultrasound. A fine-needle aspiration (FNA) biopsy was carried out for cases with an identified solitary or dominant thyroid nodule (>10mm). All surgeries were carried out by one surgical oncologist with 23 years of experience.

Surgical technique

**Figure 1:**
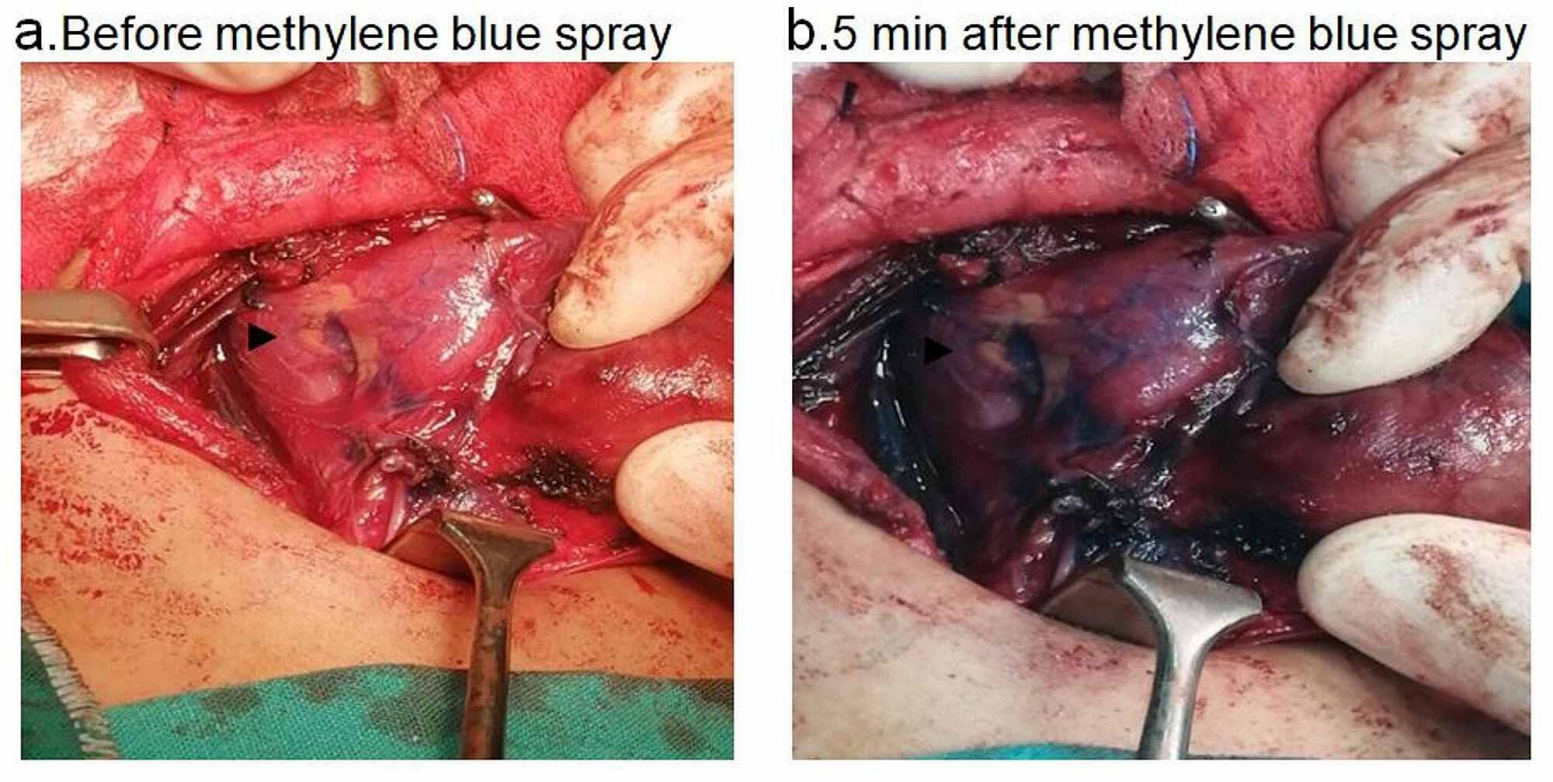
Intraoperative pictures showing parathyroid gland (marked with black arrow) before and after methylene blue dye spray.

Patients were positioned in a supine reverse Trendelenburg position (20°) with slight neck extension. Thyroidectomy was carried out through a standard transverse cervical incision following the ligation of the superior thyroid artery. Methylene blue 1% (1 ml [10 mg]) was sprayed over the thyroid lobe and perilober area, which include the parathyroid glands, the inferior thyroid artery, and the recurrent laryngeal nerve (Figure 1). Meticulous dissection and ligation of the terminal branches of the inferior thyroid artery and vein were done to preserve the vessels supplying the parathyroid glands. Subsequently, the gland was separated from the trachea, followed by an adequate inspection to ensure that no parathyroid glands were inadvertently removed. All patients had a 10f suction drain removed the following day (Figure 1).

Postoperative care

Day one postoperative clinical assessment consisted of an investigation for any complications or symptoms associated with hypocalcaemia, including perioral paraesthesia, finger numbness, cramping, signs of confusion, disorientation, delirium, seizures, Chvostek’s sign, Trousseau’s sign, as well as electrocardiogram (ECG) (long QT intervals or arrhythmia) changes. All patients had their SiCa levels checked 24h and 48h postoperatively. Hypercalcaemic patients also had their PTH levels checked 24h after surgery.

Management of postoperative hypocalcaemia

**Figure 2:**
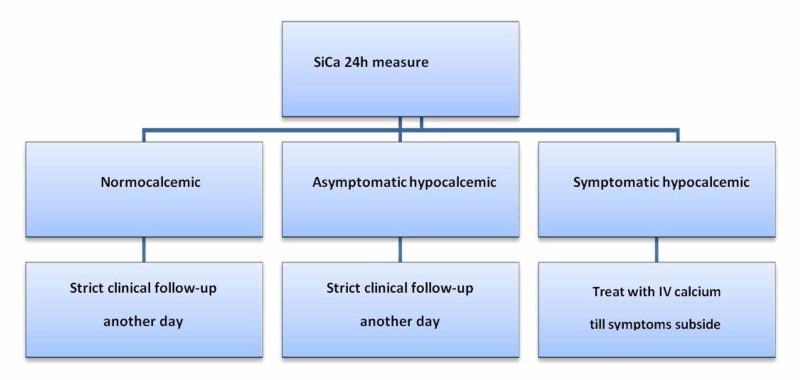
Day one postoperative serum calcium management scheme. SiCa: serum ionized calcium level

All patients had their SiCa levels measured on the first postoperative day. Normocalcaemic patients stayed another day in the hospital for clinical follow-up. Asymptomatic hypocalcaemic patients (SiCa >3.7 mg/dl) remained in the hospital for an additional day for clinical follow up without calcium supplementation. Symptomatic hypocalcaemic patients (SiCa <3.7 mg/dl) were given 10 ml 10% calcium gluconate in 100 ml of normal saline IV over 10 minutes; this procedure was repeated up to three times in patients until they became asymptomatic. Patients diagnosed with hypocalcaemia underwent maintenance therapy of 1-3 mg of calcium gluconate/kg body weight/h usually over the first 24h to 48h after surgery; then, these patients were prescribed calcium (1.5 gm/day) and vitamin D (calcitriol 1.5 μg/day) supplements (Figure 2).

All patients had their SiCa levels measured on the second postoperative day. Normocalcaemic patients were discharged without being prescribed calcium supplements.

If previously asymptomatic hypocalcaemic patients’ SiCa levels normalised, then patients were discharged with no prescription of calcium supplements. If SiCa levels remained low with no symptoms, patients were kept in the hospital for another day with strict clinical follow-up and serial SiCa level measurements in the mornings of the subsequent days until spontaneous correction. If SiCa levels showed a further decrease, and the patient developed hypocalcaemia symptoms at any time, IV calcium therapy was prescribed as described above.

**Figure 3:**
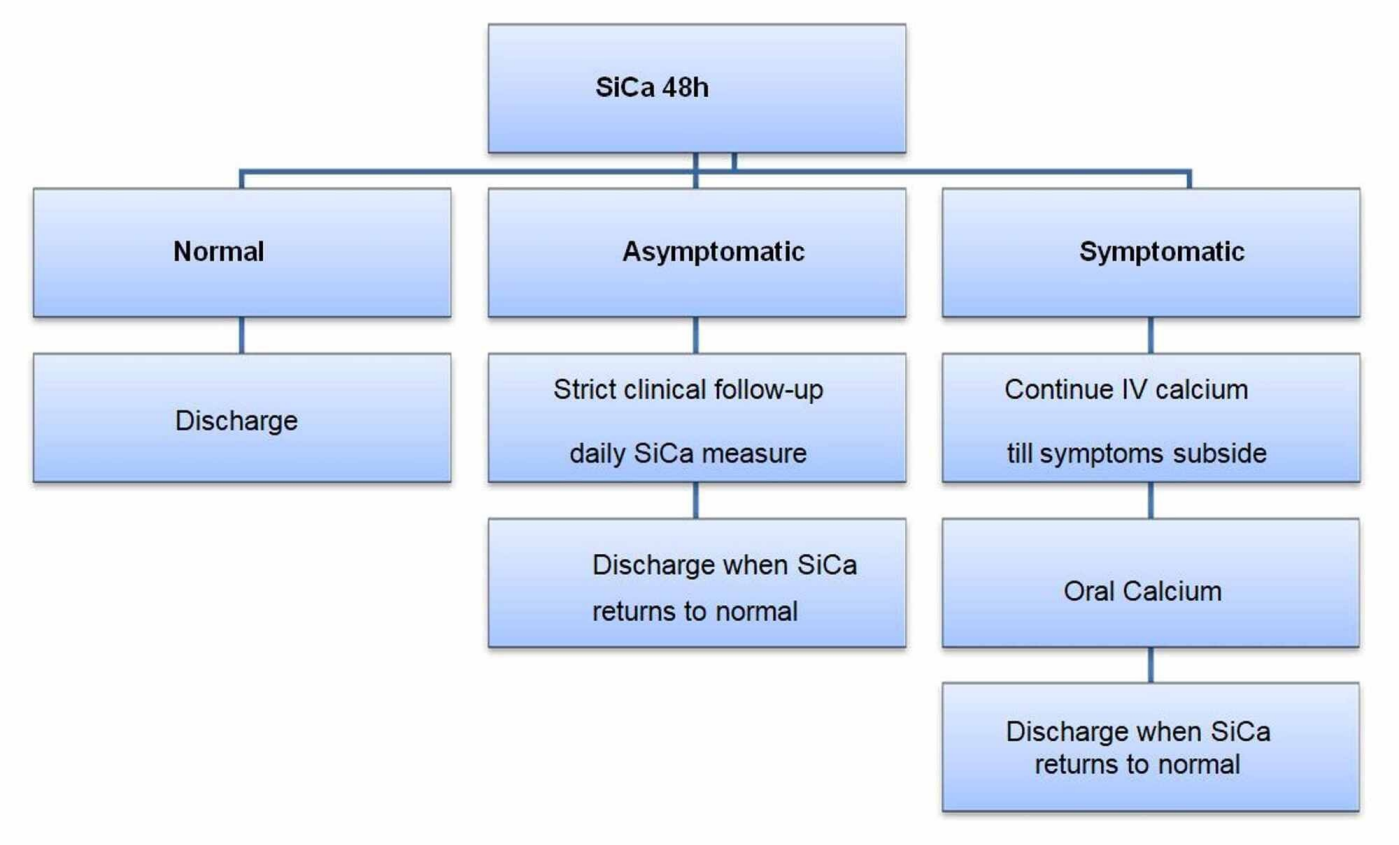
Day two postoperative serum calcium management scheme. SiCa: serum ionized calcium level

If symptoms subsided from the symptomatic hypocalcaemic patients, the IV calcium therapy was replaced by oral calcium (1.5 gm/day) and vitamin D (calcitriol 1.5 μg/day) supplements. Patients remained in the hospital with strict clinical follow-up and serial SiCa level measurements in the mornings of the subsequent days until spontaneous correction (Figure 3).

Our policy was to discharge all patients with SiCa levels within normal range (4.4 mg/dl) on the second day after surgery. Patients who met this value were discharged with clear instructions to contact our hospital any time if they developed symptoms of hypocalcaemia and to visit our outpatient clinic one week after the operation to assess the postoperative wound and clinical manifestations of hypocalcaemia, and to discuss the pathology report.

Asymptomatic hypocalcaemic patients were instructed to measure their SiCa levels one week after the operation. The patients were prescribed oral supplements if levels decreased below our reference range.

Only symptomatic hypocalcaemic patients were instructed to continue on oral calcium (1.5 gm/day) and vitamin D (calcitriol 1.5 μg/day) supplements after hospital discharge and to perform weekly SiCa level measurements for the next two to three weeks after the operation.

## Results

The total number of patients was 50, and ages ranged from 18 to 80 years old with a mean of 43, SD 9.7. Seventy percent (N=35) of patients were females, and 30% (N=15) were males. Past medical history included that seven patients (14%) were diabetic on oral hypoglycaemic medications.

We have observed that the recurrent laryngeal nerve did not take the dye at all; the wash-out time was five minutes for parathyroid glands and 15 minutes for the thyroid tissue. Following the identification of the parathyroid glands, every attempt was made to preserve them, their blood supply, as well as the recurrent laryngeal nerve (Figure 1), parathyroid glands, were successfully identified intraoperatively in 82% of patients (N=41).

Postoperative histology revealed that 94% (N=47) of patients had multinodular goitre (MNG) and 6% (N=3) had papillary carcinoma. Thyroid gland specimens showed parathyroid gland in six patients (12%) out of which four patients (8%) had one gland and two patients (4%) had two glands each, out of the six patients four patients developed postoperative asymptomatic hypocalcaemia. 

Hospital stay ranged from one to three days (1.4±0.8), during which time patients underwent clinical assessments, calcium level monitoring and treatment for hypocalcaemia. Eighteen percent of patients (N=9) developed postoperative hypocalcemia (three patients (6%) were symptomatic); postoperative serum calcium ranged from 3.5 to 5.6 (4.8±0.6), and postoperative PTH ranged from 2 to 52 pmol/L (22.2±10.1). Differences in calcium and parathyroid hormone (PTH) levels according to the parathyroid identification are shown in Table 1.

**Table 1:** Difference in calcium (Ca) and parathyroid hormone (PTH) according to parathyroid identification.

	Parathyroid identification				P-value#
	Yes (N=41)		No (N=9)		
	Mean	SD	Mean	SD	
Ca (post)	5.0	0.3	3.8	0.2	<0.0001*
PTH (post)	25.9	6.8	5.6	3.5	<0.0001*

Statistical methods

The data was organised and statistically analysed using the Statistical Package for Social Sciences (SPSS) Statistics version 22 (IBM Corp., Armonk, NY, USA). For quantitative data, the mean, standard deviation (SD), and range were calculated. The independent t-test was used to compare between the two groups of parathyroid identification as regards Ca and PTH. Qualitative data collected were presented as numbers and percentages, and the Chi-square test was used for significance. For interpretation of the results of significance tests, p-values were considered significant at p<0.05.

## Discussion

Wilhelm Fabricius performed the first thyroidectomy in 1646; however, the patient died, and the surgeon was imprisoned [11]. In 1887, Ivar Sandström illustrated the anatomic position, blood supply, and variability of the location of the parathyroid glands [12]. In 1909, McCallum and Carl Voegtlin demonstrated the relation between parathyroid glands and calcium regulation; they found that tetany following parathyroidectomy was accompanied by a calcium deficiency in tissues, and this condition could be relieved through parathyroid extract or calcium injections [13].

In 1925, Felix Mandl performed the first parathyroidectomy, but unfortunately, the patient developed recurrent hypercalcaemia and died soon after a second surgical exploration [14].

Post-thyroidectomy complications are not uncommon; risk factors attributed to thyroid surgery morbidity include the extent of resection, reoperation for completion, patient volume per surgeon and the surgeon’s experience; meticulous dissection is a key factor in minimizing complications [15,16]. Postoperative hypoparathyroidism has medical and financial implications because it leads to longer hospitalisation periods and an increase in costs.

Several methods were proposed to identify parathyroid glands in order to decrease the incidence of postoperative hypocalcaemia. Anton et al. found that frozen section has a 99% accuracy in spotting suspected parathyroid tissue; however; this is an expensive technique [3]. Suzuki et al. concluded that intraoperative photodynamic detection of normal parathyroid glands using 5-aminolevulinic acid (5-ALA) has a 100% specificity, but it is expensive, has a long test duration, and is also not available to all hospitals [5]. Huang et al. demonstrated that FNA with measurements of parathyroid hormone levels has a 97.8% sensitivity and 100% specificity in thyroidectomy; however, this method is not available to all hospitals and has high cost [6]. Sommerey et al. demonstrated that intraoperative optical coherence tomography has a 100% specificity [4]. Bewick et al. were able to identify parathyroid glands in 78.6% of cases following intravenous methylene blue; however, 5.8% of patients suffered systemic complications [7].

When Sari et al. used a methylene blue spray to locate parathyroid glands, they found that parathyroid glands can absorb the stain then regain their original yellow colour in three minutes. In comparison, thyroid tissues took 15 minutes to return to normal, and fat and muscles took over 25 minutes. The authors suggested that parathyroid glands can absorb methylene blue faster than other tissues because they have a dense lymphovascular pattern [17].

In our cohort study, we have identified parathyroid glands in about 82% of cases, with no postoperative-related or methylene blue-related complications. We also found that better results can be achieved by identifying the suspected parathyroid gland area before spraying methylene blue. In cases where the blue colour uptake is unclear, we recommend using an additional tool such as fine-needle aspiration (FNA) with parathyroid hormone level measurements to identify the parathyroid glands.

Limitations

The smaller sample size affected the statistical analysis. We are still in the process of gathering information regarding possible long-term postoperative methylene blue-related complications.

Unfortunately, only calcium level was checked preoperatively, and not parathyroid hormone levels or Vitamin D.

## Conclusions

We found that the methylene blue spray technique as a safe, cheap, and efficient method to identify the parathyroid glands during thyroidectomy, which led to minimisation of the incidence of postoperative hypocalcaemia.

Further prospective controlled randomised multicentric studies with a larger sample size should be performed to confirm the safety, reliability, and effectiveness of this technique and provide evidence-based guidelines.

In spite of the fact that we did not encounter any allergic reaction, we still recommend a skin test preoperatively to rule out any possible allergic reactions.
